# Suicide attempts in US adults with lifetime DSM-5 eating disorders

**DOI:** 10.1186/s12916-019-1352-3

**Published:** 2019-06-25

**Authors:** Tomoko Udo, Sarah Bitley, Carlos M. Grilo

**Affiliations:** 1 Department of Health Policy, Management, and Behavior, School of Public Health, University at Albany, State University of New York, 1 University Place, Rensselaer, NY 12144 USA; 2 School of Public Health, University at Albany, State University of New York, Rensselaer, 12144 USA; 3 Department of Psychiatry, Yale University School of Medicine, New Haven, CT 06510 USA

**Keywords:** Eating disorders, Anorexia nervosa, Bulimia nervosa, Binge-eating disorder, Suicide attempts, Prevalence, Epidemiology

## Abstract

**Background:**

Rates of suicide are increasing in the US. Although psychiatric disorders are associated with suicide risk, there is a dearth of epidemiological research on the relationship between suicide attempts (SAs) and eating disorders (EDs). The study therefore aimed to examine prevalence and correlates of SAs in DSM-5 EDs—anorexia nervosa (AN), bulimia nervosa (BN), and binge eating disorder (BED)—in a nationally representative sample of US adults. In addition, prevalence and correlates of SAs were examined in the two subtypes of AN—restricting (AN-R) and binge/purge (AN-BP) types.

**Methods:**

The study included 36,171 respondents in the Third National Epidemiological Survey on Alcohol and Related Conditions (NESARC-III) who completed structured diagnostic interviews (AUDADIS-5) and answered questions regarding SA histories and psychosocial impairment associated with EDs. We evaluated lifetime prevalence of SA, psychosocial impairment, clinical profiles, and psychiatric comorbidity in adults with EDs with and without SA histories, and temporal relationships between age onset of SA and EDs.

**Results:**

Prevalence estimates of suicide attempts were 24.9% (for AN), 15.7% (for AN-R), 44.1% (for AN-BP), 31.4% (for BN), and 22.9% (for BED). Relative to respondents without specific EDs, adjusted odds ratios (AORs) of SAs were significantly greater in all EDs: AN = 5.40 (95% confidence intervals [CIs] = 3.80–7.67), AN-R = 3.16 (95% CIs = 1.82–5.42), AN-BP = 12.09 (95% CIs = 6.29–23.24), BN = 6.33 (95% CIs = 3.39–11.81), and BED = 4.83 (95% CIs = 3.54–6.60). Among those with SA history, mean age at first SA and number of SAs were not significantly different across the specific EDs. SA was associated with significantly earlier ED onset in BN and BED, longer duration of AN but shorter duration of BN, greater psychosocial impairment in AN and BN, and with significantly increased risk for psychiatric disorder comorbidity across EDs. Onset of BED was significantly more likely to precede SA (71.2%) but onsets of AN (50.4%) and BN (47.6%) were not.

**Conclusions:**

US adults with lifetime DSM-5 EDs have significantly elevated risk of SA history. Even after adjusting for sociodemographic factors, those with lifetime EDs had a roughly 5-to-6-fold risk of SAs relative to those without specific EDs; the AN binge/purge type had an especially elevated risk of SAs. SA history was associated with distinctively different clinical profiles including greater risk for psychosocial impairment and psychiatric comorbidity. These findings highlight the importance of improving screening for EDs and for suicide histories.

**Electronic supplementary material:**

The online version of this article (10.1186/s12916-019-1352-3) contains supplementary material, which is available to authorized users.

## Background

In 1999–2016, the rates of suicide in the United States (US) increased in all states, except for Nevada, with more than 30% increase in 25 states, and this increasing pattern was observed regardless of sex, racial/ethnic groups, and urbanity [1]. Since 2008, suicide has remained the 10th leading cause of deaths in the US, [2] making suicide prevention an urgent public health issue. Research has increasingly focused on identifying risk factors and predictors of suicidal behaviors and suicide completion; identifying reliable variables, however, has been challenging for many reasons including methodological limitations (e.g., variability in target populations, cross-sectional vs. longitudinal, short follow-up periods) [3, 4]. Nonetheless, critical and meta-analytic reviews of the literature have identified some variables that may predict suicide risk reasonably consistently across studies [3]. For example, certain sociodemographic characteristics have been associated with increased risk for suicide, including male gender, sexual orientation, race (being Caucasian), and lower education [3, 5]. Psychiatric illnesses are also one of the strongest dynamic, malleable factors associated with suicide [3, 6]. Systematic reviews have consistently reported that approximately 90% of individuals had diagnosable psychiatric disorders at the time when they died by suicide [7, 8].

Mood disorders, substance use disorders (SUD), and anxiety disorders are the most common predictors of suicide [9–11]. In addition, analyses of psychiatric-patient samples have reported that eating disorders (EDs) also appear to be associated with suicide [12, 13]. EDs, which includes include anorexia nervosa (AN), bulimia nervosa (BN), and binge eating disorder (BED) according to the *Diagnostic and Statistical Manual of Mental Disorders, Fifth Edition* (DSM-5, [14]), are prevalent psychiatric disorders that occur across age groups, sex, and race/ethnicity [15, 16]. Preti and colleague [17] reported that among 16,342 patients with AN pooled from 40 studies, the standardized mortality ratio (SMR; i.e., mortality risk relative to age- and gender-matched general population) was 31.0 (95% CI = 21.0–44.0, mean follow-up = 11.1 years). The same study reported that among 1768 patients with BN pooled from 16 studies, SMR was 7.5 (95% CI = 1.6–11.6, mean follow-up = 7.5 years); it was not possible to calculate a SMR for BED because there were only 3 studies and none reported suicide incidence [17].

A recent meta-analysis of 2611 longitudinal studies also found that ED diagnosis was associated with significantly increased risk for suicide attempt (SA) [18] although the rate of SA varied considerably across studies. In clinic samples, the rate of SA in patients with AN ranges from 3 to 20% [19], 25–35% in BN [19], 12.5% in BED [20], and 20.8% when combining all EDs [21]. Crow et al. [22], which specifically examined suicidal behaviors in US adolescents and adults with data from the National Comorbidity Survey-Replication (NCS-R; *N* = 2980) [23] and the National Comorbidity Survey-Adolescent Supplement (NCS-A; *N* = 10,123) [24, 25], estimated that the prevalence of SA in adults was 8.8% in AN, 21.3% in BN, and 15.2% in BED, whereas it was only 1.2% in adults without a lifetime psychiatry disorder.

Collectively, these studies suggest associations between EDs and suicidal behaviors, although there remain important questions to be answered. First, because BED was not a formal specific diagnosis until DSM-5, most prior studies included BED as part of the larger and heterogeneous “eating disorder not otherwise specified” category. Research on the relationship between suicidal behaviors and BED has thus been limited relative to AN or BN. In their systematic review, Conti and colleagues [26] considered 17 original studies on the relationship between BED and suicidal behaviors; the prevalence of suicide ideation ranged from 26.3 to 51.7% and the prevalence of SA ranged 5.5 to 33.6% across the various studies. The five studies that considered BED reported significantly increased odds of suicidal behaviors relative to individuals without BED [26]; all these studies, however, were based on previous DSM-IV criteria, and most focused on patients with BED and comorbid conditions. Furthermore, besides several studies that examined comorbid psychiatric conditions [21, 27, 28], body mass index (BMI) [21, 29], and ED pathologies [21, 29] as risk factors for SA in EDs, research on correlates of suicidal behaviors in EDs has been narrow and limited.

Most previous studies on suicide and EDs were based on clinical samples, which suffer from various treatment-seeking and selection confounds [30–32]. The very few available epidemiological studies often did not include EDs, particularly those conducted based in the US. For example, there are two studies that reported significantly increased risk for suicidal attempts [10, 11] and suicidal ideation [33] in individuals with psychiatric disorders using the NCS-R and the WHO World Mental Health Surveys, but *neither* considered EDs in their analyses of suicide behaviors, making an earlier mentioned Crow et al. [22] study the only epidemiological study on the relationship between EDs (but specifically BN) and suicide risk in the US adults. Thus, there exist no epidemiological studies in the US that have reported on the relationship between DSM-5-defined EDs and suicide. A number of substantive changes in diagnostic criteria for EDs were made between DSM-IV*-TR* and DSM-5. In DSM-5, the AN diagnosis no longer requires amenorrhea and now defines low-weight as less than minimally normal for age. The BN diagnosis now has a frequency requirement of once-weekly for binge eating and weight-compensatory behaviors, which is lower than the twice-weekly frequency requirement in the DSM-IV. BED, now a “formal” diagnosis, is also defined with a lower frequency requirement of once-weekly binge eating for a duration of 3 months (less than a 6-month criterion in previous research using the DSM-IV “research definition”) to parallel the BN diagnosis. These substantive changes across the DSM-5-defined EDs dictate the need for further research on the prevalence and correlates of SA in this group.

The National Epidemiologic Survey on Alcohol and Related Conditions III (NESARC-III) [34] is the first large psychiatric epidemiology study with a representative sample of US adults since DSM-5 was published. Using the NESARC-III, the goals of the present study were to (1) obtain the prevalence estimates of SA in US adults with lifetime DSM-5 ED diagnoses, (2) examine sociodemographic, clinical, and psychiatric correlates of SA in EDs, and (3) examine temporary relationships between DSM-5 ED diagnoses and first SA. In addition to the specific ED diagnoses of AN, BN, and BED, we aimed to also examine the two subtypes of AN—restricting and binge/purging subtypes of AN (AN-R and AN-BP, respectively)—given prior findings suggesting that prevalence of suicide completion was greater for AN-BP than for AN-R [29].

## Methods

### Study sample

NESARC-III, designed originally to estimate the prevalence of alcohol use and related conditions in adults, includes 36,309 non-institutionalized US civilians 18 years and older [34, 35]. All respondents completed computer-assisted face-to-face personal interviews between April 2012 and June 2013. A multi-stage probability sampling was employed with counties or groups of contiguous counties as primary sampling units, groups of Census-defined blocks as secondary sampling units, and households within secondary sampling units as tertiary sampling units. Eligible individuals were randomly selected from each household, but Hispanic, Black, and Asian household members were oversampled (i.e., two respondents from households with more than four eligible minority members). One hundred thirty-five respondents were excluded from this study due to missing data on suicide history, resulting in a total sample size of 36,171. NERSAC-III received an approval from the National Institute of Health (NIH) Institutional Review Board (IRB) and participants provided oral informed-consent [35]. The University at Albany IRB deemed that this secondary analysis study was exempted from a full-IRB approval.

### Measures

#### Diagnostic assessment of EDs and other psychiatric disorders

The NIAAA Alcohol Use Disorder and Associated Disabilities Interview Schedule-5 (AUDADIS-5) is a structured interview developed to assess a range of DSM-5-defined psychiatric disorders and their criteria, including specific information for AN, BN, and BED diagnoses [36]. The AUDADIS-5 also generated lifetime diagnoses for mood disorders (major depressive episodes, persistent depression, and bipolar I), anxiety disorders (specific phobia, social phobia, panic disorders, agoraphobia, and generalized anxiety disorder), posttraumatic stress disorder (PTSD), SUD, AUD, drug use disorder, nicotine use disorder, personality disorders (antisocial, borderline, and schizotypal), and conduct disorder. Concordance between AUDADIS-5 and clinician-administered the Psychiatric Research Interview for Substance and Mental Disorders, DSM-5 version (PRISM-5) was fair to moderate for diagnostics (*k* = 0.24–0.72) and fair to excellent for dimensional measures of SUD, mood disorders, anxiety disorders, and PTSD (intraclass correlation [ICC] = 0.43–0.72) [37, 38]. Test-retest reliability of the AUDADIS-5 for diagnosing specific disorders ranged from fair to excellent (*k* = 0.35–0.87) for diagnosis of SUD, mood disorders, anxiety disorders, PTSD, and personality disorders and from good to excellent for dimensional measures (ICC = 0.50–0.85) [39].

These NESARC studies on the validity and reliability of the AUDADIS-5, however, did not include EDs. As we detailed in our previous paper [16], we created DSM-5-based ED categories for the analyses reported (see Additional file 1 for the coding schemes, including AN-R and AN-BP), rather than utilizing the ED diagnosis variables provided by NESARC-III after close inspection of the NESARC-III ED diagnostic and criteria variables.

In addition to ED diagnoses, the AUDADIS-5 assessed age at onset, age for most recent episode, impairment in psychosocial function due to EDs, which include (1) interference with normal daily activities, (2) serious problems getting along with others, and (3) serious problems fulfilling responsibilities. Current BMI (kg/m^2^) was calculated using self-reported height and weight. Lowest BMI was calculated using the self-reported lowest weight and self-reported current height.

#### Suicide attempts (SAs)

As part of medical history, respondents answered whether they had ever attempted suicide. Respondents who reported ever attempting suicide were also asked to report age at first SA and a total number of SAs in their lifetime^1^.

#### Sociodemographic covariates

Respondents provided information about their sociodemographic status, including age, sex, race/ethnicity (non-Hispanic White, non-Hispanic Black, Hispanic, non-Hispanic Asian/Pacific Islander, and American Indianan/Alaska Native), marital status (categorized as married or living with someone as if married, widowed/separated/divorced, or never married), and education (categorized as less than high school, high school, or GED, at least some college).

### Statistical analysis

Analyses were conducted with the Statistical Analysis System (SAS) (release 9.4, 2002–2012) and accounted for NESARC survey design by using Proc Survey procedures with Taylor series variance estimation method. We calculated weight frequencies and cross-tabulations for a lifetime SA history by lifetime AN (and for AN-R and AN-BP types), BN, and BED diagnoses, and weighted means for age of first SA and the number of SAs. Multiple logistic regressions were used to calculate adjusted odds ratios (AORs) comparing odds of ever attempting suicide by lifetime AN, AN-R, AN-BP, BN, and BED diagnoses, while adjusting for socioeconomic characteristics (age, sex, race, income, and education).

We then completed the following analyses among respondents who met criteria for AN (and for AN-R and AN-BP), BN, and BED. For each ED, weighted means, frequencies, and cross-tabulations were computed for sociodemographic variables, ED clinical profiles, and comorbid psychiatric conditions by SA history. Multiple logistic regression (for dichotomous outcomes) or analysis of covariance (ANCOVA; for continuous outcomes) was used to compare means of ED clinical profile variables and odds of diagnosis for each psychiatric disorder, adjusting for sociodemographic characteristics. Significant ANCOVA analyses were probed by Tukey-Kramer post hoc analysis while significant chi-square analyses were probed by comparing cell analysis [40, 41]. Finally, to understand the temporal relationship between suicidal attempt and ED onset, we calculated weighted frequencies of respondents with primary ED diagnosis (i.e., age of ED onset comes before the age of first suicidal attempt), primary suicidal attempt, and both being reported in the same year.

## Results

### Suicidal attempts in EDs

Prevalence of ever attempting suicide, relative to those without specific ED diagnosis, was significantly higher among individuals with lifetime AN (24.9% [3.12] vs. 5.0% [0.18]), AN-R (15.7% [3.51] vs. 5.0 [0.18]), AN-BP (44.1% [7.39] vs. 5.0 (0.18)], BN (31.4% [6.06] vs. 5.1% [0.18]), and BED (22.9% [2.58] vs. 5.0% [0.18]) (Table 1). Adjusting for sociodemographic characteristics, the odds of ever attempting suicide was significantly higher in all EDs, relative to those without a lifetime diagnosis of respective specific ED. In the total sample, lifetime all ED diagnoses were associated with a significantly greater number of SAs, relative to those without a lifetime diagnosis of the respective specific ED. When analyses were restricted to only include respondents with SA history, the number of SAs was significantly greater in those with lifetime BED relative to those without lifetime BED. No significant differences were found in the number of SAs for those with AN, AN-R, AN-BP, or BN, and there were no significant differences in age of first SA by lifetime specific-ED diagnosis.

**Table 1:** Self-reported history of suicidal attempts (SAs) by lifetime history of DSM-5 anorexia nervosa (total and subtypes), bulimia nervosa, and binge-eating disorder

	AN		AN-R		AN-BP		BN		BED	
	Yes (*n* = 276)	No (*n* = 35,895)	Yes (*n* = 190)	No (*n* = 35,895)	Yes (*n* = 86)	No (*n* = 35,895)	Yes (*n* = 92)	No (*n* = 36,079)	Yes (*n* = 318)	No (*n* = 35,853)
Reporting a history of suicidal attempts										
% (SE) *n* (population estimates)	24.9 (3.12)^a^ 60 (467,671)	5.0 (0.18) 1935 (11,639,864)	15.7 (3.51)^a^ 28 (199,926)	5.0 (0.18) 1935 (11,639,864)	44.1 (7.39)^a^ 32 (267,745)	5.0 (0.18) 1935 (11,639,864)	31.4 (6.06)^a^ 29 (203,732)	5.1 (0.18) 1966 (11,903,803)	21.8 (2.72)^a^ 64 (436,375)	5.0 (0.18) 1931 (11,671,160)
AORs	5.42^‡^		3.16^‡^		12.07^‡^		6.34^‡^		4.45^‡^	
(95% CIs)	(3.82–7.71)		(1.83–5.44)		(6.29–23.16)		(3.38–11.88)		(3.19–6.22)	
The number of SAs^1^										
Total sample	0.5 (0.10)^‡^	0.09 (0.00)	0.2 (0.05)^‡^	0.09 (0.00)	1.1 (0.28)^‡^	0.1 (0.00)	0.7 (0.24)^‡^	0.09 (0.00)	0.6 (0.11)^‡^	0.09 (0.00)
With SA history	2.2 (0.40)	1.8 (0.08)	1.6 (0.23)	1.8 (0.08)	2.6 (0.68)	1.8 (0.08)	2.5 (0.61)	1.8 (0.08)	2.8 (0.40)^†^	1.8 (0.08)
Age of first attempt^2^	23.2 (1.60)	23.8 (0.15)	24.1 (2.45)	23.8 (0.31)	22.6 (1.93)	23.8 (0.31)	22.4 (1.70)	23.8 (0.31)	25.2 (1.49)	23.8 (0.30)

Notes. *AN* anorexia nervosa, *AN-R* anorexia nervosa restricting subtype, *AN-BP* anorexia nervosa binge/purging subtype, *BN* bulimia nervosa, *BED* binge eating disorder, *ED* groups are not mutually exclusive as respondents may have had multiple lifetime ED diagnosis. Calculations of adjusted odds ratios (AORs) and 95% confidence intervals (CIs), means and associated standard errors were with adjusting for sex, age, income, education, and race/ethnicity. All analyses were adjusted for the NESARC complex survey design. ^1^ = statistical analyses were based on log-transformed variables due to distribution properties. ^2^ = analysis only included individuals with SA history. ^†^ = significantly different at *p* < .05; ^‡^ = significantly different at *p* < .01. ^a^ = significantly different from no history of ED at *p* < .05 based on or comparison of cells [40, 41]

### Sociodemographic and clinical characteristics of EDs by SA history

Across the specific EDs, there were no significant differences in most sociodemographic characteristics, with a few exceptions (Table 2). In BED, respondents with SA history were significantly younger than those with no SA history. In AN-R, the proportion of females was significantly lower in respondents with SA history, relative to those with no SA history. In AN and BN, a proportion of respondents with income ≥ $70,000 was significantly lower in those with SA history, compared with no SA history. For BN, the proportion of respondents with an income range of $40,000–$69,999 was significantly greater among those with SA history than those without SA history.

**Table 2:** Sociodemographic and clinical profiles of eating disorders by a history of suicidal attempts (SA) among individuals with lifetime diagnosis of anorexia nervosa (total and subtypes), bulimia nervosa, or binge-eating disorder

	AN		AN-R		AN-BP		BN		BED	
	SA Yes (*n* = 60)	SA No (*n* = 216)	SA Yes (*n* = 28)	SA No (*n* = 162)	SA Yes (*n* = 32)	SA No (*n* = 54)	SA Yes (*n* = 29)	SA No (*n* = 63)	SA Yes (*n* = 64)	SA No (*n* = 254)
Current age (in years)	38.7 (1.92)	42.9 (1.11)	40.5 (2.65)	43.5 (1.25)	37.3 (2.49)	41.0 (2.48)	34.2 (3.23)	39.5 (2.58)	38.9 (2.31)^†^	44.9 (1.24)
% female	89.1 (5.02)	93.6 (1.64)	86.7 (7.58)^†^	94.6 (1.67)	90.8 (1.94)	90.4 (2.62)	89.6 (6.80)	87.3 (4.41)	84.0 (4.49)	73.9 (3.10)
Race/ethnicity										
Non-Hispanic White	79.0 (7.27)	79.3 (2.72)	91.6 (3.88)	80.8 (2.84)	69.6 (10.43)	74.4 (4.34)	72.1 (7.76)	74.9 (3.41)	72.8 (5.55)	73.5 (3.37)
Non-Hispanic Black	4.0 (1.50)	2.3 (0.75)	3.7 (1.82)	2.8 (0.96)	4.2 (0.78)	0.8 (0.13)	4.0 (0.79)	10.8 (1.89)	6.1 (3.58)	9.3 (1.99)
Hispanic	3.9 (1.66)	10.0 (1.51)	1.2 (0.21)	9.1 (1.81)	6.0 (1.10)	12.7 (3.11)	15.1 (6.79)	11.8 (2.41)	14.7 (5.10)	12.5 (2.04)
Other	13.0 (7.47)	8.4 (2.21)	3.5 (3.45)	7.3 (2.13)	20.2 (11.48)	12.1 (2.31)	8.8 (4.57)	8.8 (4.57)	6.4 (3.15)	4.7 (1.84)
Education										
< High school	13.6 (4.89)	5.7 (1.40)	24.1 (8.91)	6.1 (1.65)	5.8 (4.20)	4.4 (1.67)	12.5 (3.68)	9.3 (1.42)	14.1 (4.01)	11.6 (1.95)
High school or GED	17.8 (5.71)	14.6 (2.22)	9.7 (3.76)	11.3 (2.08)	23.8 (7.27)	24.8 (4.42)	20.8 (7.03)	17.6 (2.13)	22.6 (5.52)	21.7 (2.45)
Some college+	68.6 (7.01)	79.7 (2.49)	66.1 (8.72)	82.6 (2.42)	70.4 (8.43)	70.7 (4.83)	66.7 (7.65)	73.1 (2.69)	63.3 (6.40)	66.8 (3.61)
Income level										
< $25,000	26.0 (5.50)	13.6 (2.36)	22.0 (7.22)	11.4 (2.06)	29.0 (7.24)	20.5 (6.09)	24.0 (5.34)	30.0 (3.09)	26.3 (5.38)	26.2 (2.61)
$25,000–39,999	17.2 (5.52)	11.6 (1.86)	21.0 (8.31)	10.4 (1.74)	14.4 (4.77)	15.5 (4.75)	18.0 (3.67)	13.0 (3.18)	20.3 (5.41)	16.6 (2.51)
$40,000–69,999	42.5 (7.77)	25.7 (2.78)	41.4 (8.15)	25.6 (3.11)	43.4 (9.30)	26.3 (4.09)	49.1 (8.8)^†^	20.6 (2.46)	33.9 (6.61)	23.0 (2.93)
≥ $70,000	14.2 (4.92)^†^	49.0 (3.26)	15.5 (8.09)	52.6 (3.37)	13.2 (6.13)	37.7 (5.57)	8.9 (5.20)^†^	36.4 (5.25)	19.4 (5.05)	34.2 (3.84)
Age of ED onset	18.7 (0.16)	19.0 (0.03)	19.3 (0.15)	18.2 (0.08)	18.7 (1.37)	20.2 (1.20)	18.2 (0.76)^‡^	20.3 (0.69)	23.5 (0.39)^†^	24.4 (0.39)
Years with ED episodes	13.9 (0.12)^‡^	11.4 (0.13)	12.2 (0.95)	11.3 (0.55)	14.1 (0.80)^‡^	10.5 (0.63)	12.8 (0.18)^†^	13.4 (0.17)	14.1 (1.02)	14.4 (0.29)
Current BMI	26.3 (1.03)	25.4 (0.86)	25.9 (0.49)	25.4 (0.61)	25.5 (1.60)	25.9 (1.22)	28.6 (1.35)	29.0 (0.94)	34.6 (1.36)	33.4 (0.69)
Lowest BMI	16.5 (0.25)^†^	17.3 (0.21)	16.8 (0.25)	17.0 (0.21)	16.6 (0.32)^‡^	17.5 (0.27)	21.0 (0.50)^‡^	23.1 (0.27)	23.3 (0.70)^†^	25.0 (0.47)
Interfere with normal daily activities										
% (SE)	30.2 (7.72)^†^	16.4 (2.42)	9.9 (4.69)	11.8 (2.35)	45.4 (10.14)	30.7 (4.84)	68.4 (9.68)	49.9 (5.41)	44.0 (6.19)	54.4 (3.98)
Serious problems getting along with others										
% (SE)	28.5 (8.08)	16.2 (2.67)	10.8 (4.68)	14.3 (2.80)	41.6 (10.78)	22.2 (4.65)	33.5 (8.95)	32.6 (2.91)	18.7 (6.23)	18.6 (2.78)
Serious problems fulfilling responsibilities										
% (SE)	24.8 (7.30)^†^	9.5 (1.77)	6.1 (3.98)	5.5 (1.57)	38.8 (9.92)	21.9 (3.90)	54.6 (13.25)^†^	27.7 (3.92)	17.5 (5.76)	26.8 (3.45)
Any form										
% (SE)	36.8 (8.09)	24.8 (3.20)	19.8 (6.36)	18.9 (3.18)	49.4 (10.81)	43.2 (5.87)	76.8 (8.53)	58.4 (5.75)	44.0 (6.19)	54.9 (3.98)

Notes. *AN* anorexia nervosa, *AN-R* anorexia nervosa restricting subtype, *AN-BP* anorexia nervosa binge/purging subtype, *BN* bulimia nervosa, *BED* binge eating disorder, *SA* suicide attempt. ED groups are not mutually exclusive as respondents may have had multiple lifetime ED diagnosis. Calculations of means and associated standard errors for were with adjusting for sex, age, income, education, and race/ethnicity for comparison of clinical profiles. All analyses were adjusted for the NESARC complex survey design. ^1^ = statistical analyses were based on log-transformed variables due to distribution properties. ^†^ = significantly different at *p* < .05 based on Tukey-Kramer post hoc analysis or comparison of cells [40, 41]; ^‡^ = significantly different at *p* < .01

For BN and BED, respondents with SA history reported significantly younger age of ED onset, whereas SA history was associated with significantly later ED onset for AN-R. For AN and AN-BP, SA history was associated with a significantly longer ED episode, whereas it was associated with significantly less years with ED episode in BN. In AN, AN-BP, BN, and BED, the lowest BMI was significantly lower among those with SA history than those with no history. In AN, a greater proportion of respondents with SA history reported that their ED symptoms interfere with daily normal activities and cause serious problems getting along with others, relative to those without SA history. In BN, a greater proportion of respondents with SA history reported that their ED symptoms caused serious problems getting along with others, relative to those without SA history.

### Lifetime comorbid DSM-5 psychiatric diagnosis by SA history

Table 3 summarizes prevalence of each of the DSM-5 psychiatric disorders by SA history, separately for each ED diagnosis. In the three specific EDs, even after adjusting for sociodemographic variables, SA history was associated with significantly increased odds of any mood disorder, major depressive disorder, persistent depression, any anxiety disorder, panic disorder, PTSD, any personality disorder, antisocial and borderline personality disorder, and conduct disorder (Table 4). Additionally, SA history was significantly associated with bipolar I disorder, specific phobia, any SUD, AUD, other drug use disorder, and schizotypal personality disorder in AN, with bipolar I disorder, AUD, and schizotypal personality disorder in BN, and with agoraphobia and other drug use disorder in BED.

**Table 3:** Prevalence (standard errors [SEs]) of other lifetime DSM-5 psychiatric disorders by a history of suicidal attempts (SA) among individuals with lifetime diagnosis of anorexia nervosa (total and subtypes), bulimia nervosa, or binge-eating disorder

	AN		AN-R		AN-BP		BN		BED	
	SA Yes (*n* = 60)	SA No (*n* = 216)	SA Yes (*n* = 28)	SA No (*n* = 162)	SA Yes (*n* = 32)	SA No (*n* = 54)	SA Yes (*n* = 63)	SA No (*n* = 29)	SA Yes (*n* = 64)	SA No (*n* = 254)
Any mood disorders	86.4 (5.37)	43.5 (2.82)	81.7 (7.52)	38.7 (3.32)	89.9 (7.09)	58.6 (5.55)	96.0 (2.96)	72.0 (4.57)	88.1 (3.01)	64.9 (3.09)
Major depressive disorder	83.1 (5.98)	38.4 (3.06)	74.1 (9.04)	36.0 (3.19)	89.9 (7.09)	46.0 (6.48)	96.0 (2.96)	67.3 (4.75)	88.1 (3.01)	59.3 (3.14)
Persistent depression	46.9 (7.85)	14.2 (2.05)	50.2 (8.39)	12.2 (1.89)	44.4 (9.10)	20.7 (3.05)	56.5 (9.29)	24.8 (6.18)	53.7 (6.85)	27.0 (3.05)
Bipolar I	21.3 (6.75)	3.7 (1.68)	19. (7.90)	2.8 (0.98)	22.8 (6.83)	6.5 (0.84)	32.1 (11.11)	4.3 (0.37)	24.9 (6.08)	4.2 (1.52)
Any anxiety disorders	58.8 (8.04)	34.4 (2.85)	45.1 (8.81)	33.5 (2.35)	69.1 (9.45)	37.3 (5.52)	60.5 (11.17)	37.3 (5.85)	76.1 (5.21)	54.2 (3.29)
Panic disorder	36.3 (7.73)	16.0 (2.47)	23.1 (8.61)	14.0 (1.69)	46.1 (9.75)	22.0 (3.33)	32.2 (11.09)	11.2 (2.52)	45.7 (5.83)	16.1 (2.16)
Agoraphobia	17.7 (7.74)	8.6 (2.32)	9.3 (7.21)	5.6 (1.80)	23.9 (11.27)	18.0 (3.85)	13.6 (2.46)	8.0 (2.27)	23.6 (4.78)	10.6 (1.92)
Social anxiety disorder	18.1 (5.57)	5.6 (1.35)	14.6 (7.39)	4.8 (1.07)	20.6 (3.80)	8.0 (3.80)	22.8 (6.77)	10.1 (2.18)	30.7 (5.53)	17.8 (3.00)
Specific phobia	24.7 (7.74)	12.2 (2.38)	13.7 (4.82)	12.2 (2.20)	32.9 (10.25)	12.3 (3.02)	20.8 (7.19)	11.5 (3.65)	20.5 (5.06)	25.5 (2.89)
General anxiety disorder	31.8 (7.95)	18.7 (2.39)	22.1 (7.55)	17.3 (1.52)	39.1 (10.74)	23.1 (4.52)	27.9 (9.82)	24.9 (5.63)	41.1 (6.29)	30.7 (2.69)
Posttraumatic stress disorder	49.5 (7.91)	13.8 (2.79)	37.0 (8.90)	11.3 (2.86)	58.9 (8.49)	21.5 (4.15)	57.1 (13.19)	21.2 (2.63)	58.4 (7.31)	24.1 (2.41)
Any substance use disorder	82.9 (4.80)	52.8 (3.45)	91.2 (4.58)	45.4 (4.27)	76.7 (5.84)	76.1 (4.71)	82.1 (3.74)	60.0 (5.92)	75.9 (6.04)	65.4 (3.08)
Alcohol use disorder	72.8 (5.90)	41.4 (2.76)	71.0 (8.77)	34.2 (3.23)	74.2 (6.40)	64.1 (5.81)	79.9 (4.85)	52.3 (5.79)	61.3 (6.08)	49.4 (3.29)
Nicotine use disorder	59.1 (9.01)	31.7 (3.79)	77.4 (8.40)	26.4 (4.44)	45.5 (8.93)	48.2 (6.89)	68.5 (9.97)	30.7 (3.23)	50.9 (5.87)	37.3 (3.42)
Other drug use disorder	37.1 (6.74)	11.2 (1.89)	39.8 (9.45)	6.5 (2.00)	35.1 (7.16)	25.9 (4.32)	45.2 (10.92)	22.4 (4.25)	39.0 (6.61)	20.8 (2.30)
Any personality or conduct disorder	73.3 (8.47)	22.5 (2.76)	57.3 (8.19)	16.9 (2.60)	85.2 (5.78)	40.4 (5.42)	78.7 (14.13)	38.4 (3.90)	81.5 (4.83)	48.9 (3.48)
Antisocial	18.6 (5.61)	5.5 (1.81)	16.4 (6.74)	2.2 (0.97)	20.1 (4.85)	15.9 (3.63)	22.5 (7.17)	5.2 (1.89)	30.7 (5.65)	11.7 (2.10)
Borderline	62.1 (8.86)	19.7 (2.79)	48.6 (8.50)	13.8 (2.44)	72.2 (8.94)	38.4 (5.45)	77.2 (14.01)	34.6 (4.06)	77.6 (4.90)	41.3 (3.77)
Schizotypal	40.7 (7.71)	9.1 (2.04)	24.7 (8.24)	5.2 (1.19)	52.6 (9.62)	21.1 (4.35)	40.3 (9.61)	17.4 (3.23)	36.9 (5.70)	25.3 (3.05)
Conduct	24.3 (6.26)	5.7 (1.82)	29.9 (9.35)	2.5 (1.02)	20.1 (4.85)	15.9 (3.63)	22.5 (7.17)	8.7 (1.99)	30.7 (5.65)	12.0 (2.12)

Notes. *AN* anorexia nervosa, *AN-R* anorexia nervosa restricting subtype, *AN-BP* anorexia nervosa binge/purging subtype, *BN* bulimia nervosa, *BED* binge eating disorder. ED groups are not mutually exclusive as respondents may have had multiple lifetime ED diagnosis. All analyses adjusted for complex survey design of NESARC-III

**Table 4:** Adjusted odds ratios (AORs) and 95% confidence intervals (95% CIs) of other psychiatric disorders by a history of suicidal attempts among individuals with lifetime diagnosis of anorexia nervosa (total and subtypes), bulimia nervosa, or binge eating disorder

	AN	AN-R	AN-BP	BN	BED
Any mood disorders	*8.60* (3.13–23.64)^‡^	*6.74* (1.95–23.26)^‡^	*8.32* (1.72–40.15)^†^	*13.30* (1.71–103.34)^†^	*3.49* (1.77–6.88)^‡^
Major depressive disorder	*9.03* (3.36–24.28)^‡^	*6.17* (2.06–18.46)^‡^	*15.06* (2.67–84.89)^‡^	*14.39* (2.05–100.99)^‡^	*4.32* (2.18–8.55)^‡^
Persistent depression	*5.29* (2.47–11.35)^‡^	*5.55* (2.54–12.09)^‡^	*3.63* (1.27–10.35)^†^	*7.68* (2.82–20.90)^‡^	*3.36* (1.82–6.20)^‡^
Bipolar I	*5.42* (1.30–22.64)^†^	*6.56* (1.97–21.80)^‡^	1.73 (0.36–8.40)	*25.40* (10.96–58.87)^‡^	*6.73* (2.58–17.55)^‡^
Any anxiety disorders	*2.60* (1.27–5.33)^†^	1.63 (0.75–3.56)	*3.48* (1.34–9.03)^†^	*3.78* (1.16–12.34)^†^	*2.72* (1.39–5.32)^‡^
Panic disorder	*2.38* (1.19–4.57)^†^	1.23 (0.57–2.69)	*2.07* (1.04–4.10)^†^	*3.68* (1.34–10.09)^†^	*4.21* (2.33–7.60)^‡^
Agoraphobia	2.03 (0.66–6.27)	–	1.07 (0.29–3.99)	–	*2.88* (1.45–5.74)^‡^
Social anxiety disorder	2.06 (0.80–5.32)	1.71 (0.56–5.21)	2.50 (0.53–11.86)	–	*2.02* (1.04–3.92)^‡^
Specific phobia	*2.64* (1.00–6.96)^†^	1.15 (0.45–2.97)	*3.26* (1.17–9.06)^†^	*3.86* (1.66–9.00)^‡^	0.73 (0.34–1.54)
General anxiety disorder	1.99 (0.91–4.37)	–	2.03 (0.61–6.76)	2.02 (0.62–6.56)	1.67 (0.91–3.09)
Posttraumatic stress disorder	*5.71* (2.73–11.92)^‡^	*4.78* (1.80–12.70)^‡^	*4.88* (2.22–10.70)^‡^	*12.84* (4.70–35.06)^‡^	*4.27* (2.02–8.99)^‡^
Any substance use disorder	*3.33* (1.65–6.71)^‡^	*10.38* (2.84–37.85)^‡^	0.83 (0.29–2.32)	*2.26* (1.13–4.51)^†^	1.64 (0.78–3.47)
Alcohol use disorder	*3.53* (1.82–6.84)^‡^	*6.27* (2.33–16.89)^‡^	1.55 (0.56–4.27)	*3.05* (1.50–6.24)^‡^	1.63 (0.93–2.86)
Nicotine use disorder	2.24 (0.90–5.55)	*7.70* (2.59–22.92)^‡^	0.45 (0.13–1.50)	4.01 (0.97–16.49)	1.45 (0.84–2.50)
Other drug use disorder	*4.59* (2.30–9.14)^‡^	*12.57* (3.12–50.68)^‡^	1.37 (0.48–3.89)	2.41 (0.74–7.84)	*2.17* (1.15–4.10)^†^
Any personality or conduct disorder	*6.85* (2.84–16.53)^‡^	*4.52* (1.97–10.37)^‡^	*7.80* (3.06–19.86)^‡^	*15.49* (3.30–72.67)^‡^	*4.82* (2.29–10.16)^‡^
Antisocial	*3.17* (1.21–8.30)^†^	*15.45* (2.29–104.00)^‡^	1.21 (0.38–3.80)	*11.39* (2.94–44.08)^‡^	*4.15* (2.09–8.22)^‡^
Borderline	*4.54* (1.93–10.68)^‡^	*3.52* (1.45–8.57)^‡^	3.36 (0.99–11.38)	*11.89* (3.22–43.97)^‡^	*4.93* (2.33–10.46)^‡^
Schizotypal	*6.32* (2.45–16.32)^‡^	*4.60* (1.86–11.37)^‡^	*3.90* (1.22–12.53)^†^	*3.72* (1.14–9.60)^‡^	1.61 (0.84–3.11)
Conduct	*4.27* (1.68–10.87)^†^	*20.45* (3.52–118.96)^‡^	1.21 (0.38–3.80)	*5.41* (1.59–18.46)^†^	*3.99* (2.01–7.90)^‡^

Notes. *AN* anorexia nervosa, *AN-R* anorexia nervosa restricting subtype, *AN-BP* anorexia nervosa binge eating/purging subtype, *BN* bulimia nervosa, *BED* binge eating disorder. ED groups are not mutually exclusive as respondents may have had multiple lifetime ED diagnosis. All analyses were adjusted for age, sex, race/ethnicity, and education, and complex survey design of NESARC-III. Italic letters indicate significant AORs. ^†^ = significant at *p* < .05; ^‡^ = significant at *p* < .01. – indicates invalid model estimates

In AN-R and AN-BP, SA history was significantly associated with any mood disorders, major depressive disorder, persistent depression, PTSD, any personality disorder, and schizotypal disorder. Additionally, in AN-R, SA history was significantly associated with bipolar I disorder, any SUD, AUD, nicotine use disorder, other drug use disorder, antisocial personality disorder, borderline personality disorder, and conduct disorder, whereas SA history was significantly associated with any anxiety disorder, panic disorder, and specific phobia in AN-BP.

### Temporal relationship between EDs and SA

The majority of respondents with BED reported that their onset of BED preceded their first SA (71.2% [4.47]), which was significantly different from 50% based on 95% confidence limits (Table 5). The proportions of primary AN and primary SA were similar (50.4% [6.87] vs. 44.1% [6.73]). In AN-R, a little less than half (44.4% [3.04]) reported their ED onset was earlier than their first SA whereas in AN-BP, a little more than half (54.9% [6.35]) reported their ED onset was earlier than their first SA. In BN, nearly half (47.6% [10.6]) reported that their BN onset preceded their first SA, and about one third of BN (34.9% [11.7]) reported that their onset of BN and first SA occurred within the same year.

**Table 5:** Temporary relationships between onsets of DSM-5 eating disorders and suicide attempts

	AN (*n* = 60)	AN-R (*n* = 28)	AN-BP (*n* = 32)	BN (*n* = 29)	BED (*n* = 64)
ED onset first (% [SE]) 95% confidence limits	50.4 (6.87)	44.4 (3.04)	54.9 (6.35)	47.6 (10.6)	71.2 (4.47)^†^
	(35.3–65.5)	(34.7–54.0)	(37.2–72.5)	(18.3–76.9)	(61.6–80.8)
SAs first (% [SE]) 95% confidence limits	44.1 (6.73)	43.6 (3.24)	44.5 (6.32)	17.5 (2.41)	26.6 (4.65)
	(29.3–58.9)	(33.3–53.9)	(27.0–62.0)	(10.9–24.2)	(16.6–36.6)
Both in the same year (% [SE]) 95% confidence limits	5.5 (1.71)	12.1 (4.02)	0.63 (0.04)	34.9 (11.7)	2.2 (0.70)
	(1.7–9.3)	(0.00–24.8)	(0.51–0.75)	(2.3–67.5)	(0.7–3.7)

Notes. *AN* anorexia nervosa, *AN-R* anorexia nervosa restricting subtype, *AN-BP* anorexia nervosa binge eating/purging subtype, *BN* bulimia nervosa, *BED* binge eating disorder. ED groups are not mutually exclusive as respondents may have had multiple lifetime ED diagnosis. All analyses were adjusted for age, sex, race/ethnicity, and education, and complex survey design of NESARC-III. ^†^ = significantly different from 50% at *p* < .05

## Discussion

Using the data from the 2012–2013 NESARC-III, this was the first study to examine the prevalence of SAs in a representative sample of the US adults with lifetime DSM-5 ED diagnoses. SAs are common in US adults with lifetime AN, BN, and BED diagnoses. We generated the following prevalence estimates for SAs across the ED diagnosis: 24.9% for AN, 31.4% for BN, and 22.9% for BED; importantly, for AN, the prevalence rate for SAs was substantially higher for the AN-BP subtype (44.1%) than for the AN-R subtype (15.7%). Adjusting for sociodemographic variables, compared with those without lifetime specific-ED diagnosis, odds of SAs were 5.40, 6.33, and 4.83 times higher in respondents with lifetime AN, BN, and BED, respectively; adjusted odds of SAs calculated separately for AN-BP and AN-R subtypes were 12.09 and 3.16, respectively. The total number of SAs was also significantly greater in respondents with lifetime specific-ED diagnosis. In those with SA history, reported age at first SA or the number of SAs were not significantly different by lifetime ED diagnoses, except for greater number of SAs in BED.

This study also attempted to address possible implications of comorbidity among different EDs as well as diagnostic cross-over [42–44] in a series of supplemental analyses. We found that the odds of SAs, although remaining statistically significant, decreased for BN and BED when we applied the hierarchical convention of AN>BN>BED for assigning ED diagnoses (Additional file 2). Among respondents who met more than one lifetime ED diagnosis, the prevalence and odds of SAs were significantly elevated (41.5% and AOR = 10.63, respectively), relative to respondents without lifetime history of specific EDs (Additional file 3). These findings are similar to those previously reported by Pisetsky et al. [29] that the highest odds of suicidal behaviors were found in those who met criteria for AN and BN. We note, however, that a more recent larger cohort study with treatment-seeking adults from the Swedish registry (*N* = 9622) found that transition between AN, BN, and BED was rare [45]. Our study also found that the comorbidity rate for different lifetime ED diagnosis was only 12% of those who met lifetime diagnosis of any EDs. Collectively, these findings suggest that while comorbidity or transition across ED diagnoses might be less common than clinically presumed, those individuals who do transition between different ED diagnoses may be at higher risk for suicide attempts.

Prevalence of SAs in our sample of US adults with lifetime DSM-5-defined EDs was higher than that of previously reported by Crow et al. [22] based on the smaller NCS-R which used DSM-IV definitions (8.8% in AN, 21.3% in BN, and 15.2% in BED). The reasons for differences are not clear and may be due partly to several methodological differences between the two studies, in addition to the many complexities inherent in assessing and generating diagnostic estimates in large epidemiological studies [46]. One notable difference between two studies is that while a question of ever attempting suicide was asked for all NESARC-III respondents, in the NCS-R this question was asked only when respondents endorsed suicidal ideation. Given the two surveys were conducted over 10 years apart, it is also possible that some of the difference partly reflect general increases in prevalence of suicide in the US [1, 47].

Within each ED category, there were only a few significant differences in sociodemographic characteristics between those who have ever versus never attempted suicide, a pattern that differs somewhat from in the general population [5]. Nonetheless, there were several significant differences in clinical characteristic between those with and without SA histories. In BN and BED, SA history was associated with earlier age of ED onset, which is partially consistent with findings from a clinical sample [21]. In all EDs, the lowest BMI was lower in respondents with a SA history than those without SA history. SA history was significantly associated with longer duration (more years) of ED episodes in AN, whereas it was associated with significantly shorter duration of ED episodes in BN. These findings, which require replication and extension in future studies, are curious and a challenge to explain. One possible clinical speculation is that SA in AN might perhaps be related to chronicity whereas SA in BN might be more related to severity. Given the relatively small sample sizes when focusing only on respondents with ED diagnoses, our study was likely underpowered to adequately statistically examine such potential reasons. We also found that among those with lifetime AN, the proportion of those reporting that ED symptoms interfered with normal daily activities or caused serious problems fulfilling responsibilities was higher among those with SA history. In addition, among those with lifetime BN, the proportion of those reporting that ED symptoms caused serious problems with fulfilling responsibilities was higher among those with SA histories. Among those with BED, the prevalence of psychosocial impairments did not differ significantly by SA history.

Consistent with previous studies [21, 22, 27, 28], SA history was significantly associated with lifetime diagnoses of other psychiatric disorders. Mood disorders, SUD, and personality disorders were prevalent across all EDs with a history of SA, with 73–96% meeting criteria for those diagnoses. Prevalence of anxiety disorders was also high in BED, with over 70% meeting criteria for diagnosis at some point in their lifetime. Within each specific ED, odds for all comorbid psychiatric disorders, even after adjusting for sociodemographic characteristics, were substantially elevated in those with SA history relative to those without SA history. This was particularly prominent in BN where many of the significant AORs were greater than 10. Further research, particularly with longitudinal designs, is necessary to replicate our findings and to examine how elevated psychiatric comorbidity in BN may contribute to future SAs as well as to other negative health and psychosocial outcomes.

In terms of sequence of ED and SA onsets, we found that for the majority of respondents with BED (71.2%), BED onset preceded the first SA. In contrast, among respondents with AN and BN, approximately 50% reported ED onset prior to the first SA. The age of onset is significantly later in BED than AN and BN [16], and the observed difference between the three forms of ED diagnoses may reflect differences in the clinical course. Our finding on BED onset preceding SA onset contrasts with a study based on the NCS-R that found that their adult respondents (in contrast to their findings for adolescents) reported SA prior to onset of BED [48]. We, however, note that due to missing data, that sample size in the Forest et al. study was extremely small (*n* = 1 for adults 18–29, *n* = 3 for 30–44, and *n* = 3 for 45–59, and *n* = 1 for 60+ years old), suggesting need for caution in interpreting those findings.

Our findings suggest that many individuals, particularly those with BED, report experiencing SAs prior to the onset of their ED symptoms. This is consistent with a recent meta-analysis by Smith [18], suggesting that while ED diagnosis and disordered eating are significantly associated with SA, they are only weak predictors of SA. Given high comorbidity between EDs and other psychiatric disorders, it is possible that the relationship between EDs and SA may be mediated by mood disorders and/or SUD, which are established significant predictors of SA [33]. Our study was cross sectional and did not investigate the temporal relationship between EDs, other psychiatric disorders, and SAs. Our findings and the findings from the prior studies highlight the need for further research specifically focused on the relationship between ED and suicide behaviors. With these caveats in mind, we state that our findings of significantly increased risk for SAs in those with lifetime EDs and that ED onset preceded first SAs in roughly half of ED cases highlights the importance of assessing and closely monitoring ED symptoms along with other psychiatric disorders by health-care providers and clinicians.

Our study also found several important differences between the two AN subtypes. We found that 32.3% (SE = 2.88) of those with lifetime AN-BP (prevalence among the total NESARC sample: AN-R = 0.54% [SE = 0.05], AN-BP = 0.26% [SE = 0.03]). Thus, when considered in this manner, across the EDs included in this study, AN-BP had the highest risk for SA whereas AN-R had the lowest, albeit significantly elevated. Our findings regarding especially elevated risk of SA in AN-BP are consistent with previous findings [29, 49].

AN-R and AN-BP subtypes of AN also had several different patterns of significant associations between SA history and other psychiatric disorders, though interpretations of those “correlates” are more complicated due to various reasons, including the fact that it is not possible to examine the complex patterns of timing associated with all the lifetime psychiatric comorbidities. With that caveat, we cautiously note some overall differences in the lifetime comorbidity findings associated with SA for the AN subtypes. In AN-R, SA history was associated with significantly elevated risk for bipolar I disorder, all forms of SUDs, and all forms of personality/conduct disorders. In contrast, in AN-BP, SA history was associated with any anxiety disorder, panic disorder, and specific phobia. Clinically, AN-BP is thought to be associated with higher impulsivity and substance use than AN-R [49–51], although differences between naturalistic, epidemiological, and treatment-seeking patterns of comorbidity may make comparisons difficult. Treatment-seeking confounds and/or motivations might, for example, partly explain why clinically AN-BP and BN show greater impulsivity and problems such as SUD, in that those externalizing and florid problems lead to seeking help as opposed to the restriction/control leading to decreased help-seeking in those with AN-R. Thus, it is possible that in naturalistic samples, SA history is associated with increased risk for SUD in those persons with AN-R. Lastly, we note that the risk for major depressive disorder was especially elevated in AN-BP with SA histories, with observed AORs similar to observed in BN.

Our findings regarding elevated risk for SA in AN-BP and BN are consistent with findings reported by Pisetsky et al. [29] that the highest rates of suicide completion occurred in those forms of EDs involving purging behaviors (comorbid AN and BN, AN-BP, BN, and purging disorder). The associations between SA and major depressive disorder were significantly increased in all EDs, and they were also particularly increased in AN-BP and BN. Thus, it is possible that the elevated risk for SAs in these forms of EDs may be partly influenced by depression. Diagnostic and clinical utility of AN subtypes has long been debated as clinical research did not always support predictive validity (e.g., [49, 51]). Our findings, however, suggest that two AN subtypes (AN-BP and AN-R) differ significantly in terms of SA risk and correlates of SA; these findings support the DSM-5 retention of the subtyping scheme.

Strengths of the current study include use of the largest psychiatric epidemiology survey on a representative sample of US adults and that all psychiatric disorders were assessed with a lay-administered structured interview. A possible limitation is that the validity and reliability of the AUDADIS-5 has not been tested for EDs, although it has been tested for all other psychiatric conditions and the findings are adequate [37, 39]. This is a cross-sectional study that retrospectively assessed lifetime history of ED symptoms and thus is susceptible to recall bias, although use of a structured diagnostic interview might have attenuated such effects to some degree. Due to the patterns of skip-out questions and missing data, we were unable to include subthreshold BN and BED or otherwise specified feeding and eating disorders (OSFED). There are other possible assessment limitations with the AUDADIS-5 that might have contributed to lower prevalence of EDs in general in this study than other reports [16, 46]. Previous studies on EDs and suicidal behaviors have also investigated the relationship between SA and specific eating pathologies, such as compensation methods (e.g., vomiting, purging) [21, 22, 29], but we were unable to explore such possible relationship with SAs due to the nature of the AUDADIS-5 questions for BN or BED [16, 52]. Relative to AN and BED, the sample size of respondents with lifetime BN diagnosis was smaller, which may have contributed to some non-statistically significant results (e.g., AORs for risk for SAs in those with and without lifetime BN diagnosis); the smaller sample size also precluded us from investigating possible sex or ethnic/racial differences. Similarly, we were unable to obtain stable estimates of the age-cohort effects on the relationship between EDs and SA, which has been recommended when investigating lifetime prevalence of psychiatric disorders with a mix-age sample [53, 54].

## Conclusions

Given that suicide remains a major cause of deaths in the US, it is crucial to develop better approaches for earlier identification and recognition of risk, which may, in turn, enhance suicide prevention strategies across the lifespan and across different clinical groups that might differ in their suicide risk. Our findings suggest that adults with a lifetime diagnosis of EDs are at substantially elevated risk for SA and suggest that health-care workers consider these findings in their screening and evaluation protocols. The prevalence of SAs in our epidemiological sample was similar or greater than those reported for clinical samples [19–21]. This is concerning as treatment utilization is very low among adults with diagnosable EDs in the general population, particularly among men and ethnic/racial minorities [30–32, 55]. Thus, routine screening for EDs along with SA history by health-care providers could inform comprehensive treatment planning and appropriate treatment referrals.

## Additional files

Additional file 1: **Table S1.** Operationalization of criteria for DSM-5 eating disorders and related entities in the AUDADIS-5 in the NESARC-III. (DOCX 31 kb)

Additional file 2: **Table S2.** Self-reported history of suicidal attempts (SAs) by lifetime history of DSM-5 anorexia nervosa (AN), bulimia nervosa (BN), binge-eating disorder (BED) (with application of hierarchical rule AN>BN>BED), and no specific eating disorder (ED) diagnosis. (DOCX 20 kb)

Additional file 3: **Table S3.** Comparison of self-reported history of suicidal attempts (SAs) between respondents meeting more than one lifetime eating disorder (ED) diagnosis and no history of specific ED diagnosis. (DOCX 19 kb)

## Data Availability

The NESARC-III data is available upon approval by the NIAAA (https://www.niaaa.nih.gov/research/nesarc-iii).

## Abbreviations

AN: Anorexia nervosa
AN-BP: Anorexia nervosa - binge/purge type
ANCOVA: Analysis of covariance
AN-R: Anorexia nervosa - restricting type
AOR: Adjusted odds ratio
AUD: Alcohol use disorder
AUDADIS-5: Alcohol Use Disorder and Associated Disabilities Interview Schedule-5
BED: Binge-eating disorder
BMI: Body mass index
BN: Bulimia nervosa
CI: Confidence interval
DSM-5: Diagnostic and Statistical Manual of Mental Disorders, Fifth Edition
DSM-IV: Diagnostic and Statistical Manual of Mental Disorders, Fourth Edition
ED: Eating disorder
GED: General educational development
ICC: Intraclass correlation
IRB: Institutional Review Board
NCS-A: National Comorbidity Survey-Adolescent Supplement
NCS-R: National Comorbidity Survey-Replication
NESARC-III: National Epidemiologic Survey of Alcohol and Related Conditions III
NIH: National Institute of Health
OSFED: Otherwise Specified Feeding and Eating Disorders
PRISM-5: Psychiatric Research Interview for Substance and Mental Disorders, DSM-5 version
PTSD: Posttraumatic stress disorder
SA: Suicide attempt
SMR: Standardized mortality ratio
SUD: Substance use disorders
US: United States

## Footnotes

1 In the original data set, respondents who reported no SA history were assigned missing for the number of SA, which we re-coded to zero for the purpose of the analyses.
