# Supplementary material for: Suicide attempts in US adults with lifetime DSM-5 eating disorders
Source: BMC Med. 2019 Jun 25;17:120. doi: 10.1186/s12916-019-1352-3 (PMC6591971; doi:10.1186/s12916-019-1352-3)
Supplement: Supplementary file 1 — Table S1. Operationalization of criteria for DSM-5 eating disorders and related entities in the AUDADIS-5 in the NESARC-III. (DOCX 31 kb) [file 12916_2019_1352_MOESM1_ESM.docx]

**Table S1.** Operationalization of criteria for *DSM-5* eating disorders and related entities in the AUDADIS-5 in the NESARC-III.

| ***DSM-5* Criteria** | **Item number** | **The AUDADIS-5 questions and operationalization in our study** |
| --- | --- | --- |
| *Current BMI* |  | Weight (in pounds) / Height (inches)^2^ x 703 |
| Current weight & height | NFEET, NINCHES, & NPOUNDS | Please tell me your height (feet & inches) and weight in pounds as these are important factors for this survey. |
| *Anorexia Nervosa* | | |
| Lowest weight | N17Q1  N17CK171^1^  (question for the interviewer) | What has been your LOWEST weight in pounds since you Weight reached your current height, not counting times when you were ill?  Is lowest weight in N17Q1 less than 85% of that expected (refer to norms for men and women)? If “NO”, skip to the next section with “Eating and Overeating.”  Operationalization in our study   - Lowest BMI calculated based on the current height - Cut-off for significantly low weight: BMI < 18.5^2^ (lowest BMI for lifetime and additionally current BMI for 12-months diagnosis) |
| A. Restriction of energy intake relative to requirements leading to a significantly low body weight in the context of age, sex, developmental trajectory, and physical health. Significantly low weight is defined as a weight that is less than minimally normal or, for children and adolescents, less than that minimally expected. | N17Q4A^3^ | When your weight was (weight in 1), did you restrict the amount of food you ate in order not to gain any weight even though other people thought you should?   - Respond “YES” |
| B. Intense fear of gaining weight or becoming fat, or persistent behavior that interferes with weight gain, even though at a significantly low weight. | N17Q5 | During that time when your weight was (N17Q1), were you afraid of gaining weight or getting fat?   - Respond “YES” |
| C. Disturbance in the way in which one's body weight or shape is experienced, undue influence of body weight or shape on self-evaluation, or persistent lack of recognition of the seriousness of the current low body weight. | N17Q6A  N17Q6B  N17Q6C  N17Q6D  N17Q6E | When your weight was (weight in N17Q1), …  Did you think that you looked fat?  Did you think your weight or body shape was one of the most important things about you?  Did you think that your weight might have been unhealthy?  Did you believe other people who thought your weight was unhealthy?  Were you constantly weighing yourself or taking measurements of various parts of your body?   - Report positive response to one of the response. Positive response is defined as “YES” for N17Q6A, N17Q6B, & N17Q6E, and “NO” for N17Q6C & N17Q6D.^4^ |
| Binge-eating/purging (or restricting) subtype | N17Q7A  N17Q7B  N17Q7D | Now I’d like to know if you did any of the following things to keep from gaining weight AFTER you reached (N17Q1)…  During either of those times did you…  Eat an UNUSUALLY LARGE amount of food within a 2-hour period, not including the holidays; that is, eat much more food than most people would eat during a 2-hour period under similar circumstances?  Vomit or use enemas, laxatives, diuretics or other medicines AFTER you ate an UNUSUALLY LARGE amount of food?  Vomit or use enemas, laxatives, diuretics or other medicines AFTER you ate a SMALL amount or REGULAR amount of food?   - Respond “YES” to one or more items - Respond “NO” to all was categorized as “Restricting” subtype |
| Age of onset | N17Q9 | About how old were you when you FIRST weighed less than (*85% of expected weight*) and had SOME of the other experiences you mentioned at the same time? |
| Age at the most recent episode | N17Q12R | About how old were you the MOST RECENT time when you weighed less than (85% of expected weight) and you also had SOME of these other experiences? |
| Duration of the most recent episode | N17Q14R | How long did (this/your) MOST RECENT time last when you weighed less than (85% of expected weight) (in weeks)?  Our operationalization of “12-months” diagnosis^5^   - Difference between age of onset and current age is 1 year or less. - Difference between current age and age at the most recent episode plus duration of the most recent episode is 1 year or less. - If we cannot calculate one of these variables, it was treated as no 12-months diagnosis |
| Associated impairments | N17Q8A  N17Q8B  N17Q8C  N17Q8D | Now, I’d like to ask you about some other things that might have happened to you during that time when you weighed (weight in N17Q1) and you had some of the other experiences we just talked about. During that time did your low weight…  Make you very upset?  Interfere with your normal daily activities?  Cause any serious problems getting along with other people – like arguing with your friends, family, people at work or anyone else?  Cause any serious problems doing the things you were supposed to do – like working, doing your schoolwork, or taking care of your home or family? |
| *Recurrent Binge Eating* | | |
| A. Eating in a discrete amount of time (e.g., within a 2 hour period) an amount of food that is definitely larger than what most individuals would eat in a similar period of time under similar circumstances. | N18Q1 | Have you EVER eaten an UNUSUALLY LARGE AMOUNT of food within any 2-hour period, not including the holidays? That is, eating more food than most people would eat during a 2-hour period under similar circumstances?   - Respond “YES” |
| B. Sense of lack of control over eating during an episode. | N18Q3a | During ANY time like this when you ate an UNUSUALLY LARGE AMOUNT of food, did you feel that you couldn’t stop eating or control how much or what you were eating?   - Respond “YES” |
| C. Binge eating occurs, on average, at least once a week for 3 months | N18Q2 | Was there EVER a time when you ate an UNUSUALLY LARGE AMOUNT of food on average at least once a week for at least 3 months?   - Respond “YES” |
| *Bulimia Nervosa* | | |
| A. Report recurrent binge eating |  | See above section |
| B. Recurrent inappropriate compensatory behavior in order to prevent weight gain such as self-induced vomiting; misuse of laxatives, diuretics, or other medications; fasting; or excessive exercise | N18Q4A | During ANY of those times when you were eating an UNUSUALLY LARGE AMOUNT of food, did you try to keep from gaining weight by vomiting, using enemas, laxatives, diuretics or other medicines, or by fasting, that is having no solid food, or exercising a lot?   - Respond “YES” |
| C. The binge eating and inappropriate compensatory behaviors both occur, on average, at least once a week for 3 months | N18Q5 | Did you EVER eat an UNUSUALLY LARGE AMOUNT of food within a 2-hour period AND do SOME of the other things we talked about to keep from gaining weight on average at least once a week for at least 3 months?   - Respond “YES” |
| D. Self-evaluation is unduly influenced by body shape and weight | N18Q3B  N18Q6 | During ANY time like this when you ate an UNUSUALLY LARGE AMOUNT of food, did you feel that your weight or body shape was one of the most important things about you  When you were eating an UNUSUALLY LARGE AMOUNT of food AND doing some of the things we talked about to keep from gaining weight around the same time, was your weight or body shape the most important thing about you?   - Respond “YES” to one of the questions |
| E. The disturbance does not occur exclusively during episodes of anorexia nervosa. |  | ***This question was not considered in this study as in many epidemiological studies with lifetime and 12-months-estimate, accurately determining whether one disorder overlap with another is not possible. |
| Age of onset | N18Q8A | About how old were you the FIRST time you BEGAN to eat LARGE AMOUNTS of food (AND do some things to keep from gaining weight) on average at least once a week for at least 3 months? |
| Age at the most recent episode | N18Q10R | How old were you the MOST RECENT time you BEGAN to eat LARGE AMOUNTS of food (AND do some things to keep from gaining weight)? |
| Duration of the most recent episode | N18Q12AR | How long did (this/your) MOST RECENT time last when you ate LARGE AMOUNTS of food (AND did OR some things to keep from gaining weight)?  Our operationalization of “12-months” diagnosis^3^   - Difference between age of onset and current age is 1 year or less. - Difference between current age and age at the most recent episode plus duration of the most recent episode is 1 year or less. - If we cannot calculate one of these variables, it was treated as no 12-months diagnosis |
| Associated impairments | N18Q7A  N18Q7B  N18Q7C  N18Q7D | Now I’d like to ask you about some other things that might have happened to you when you were eating an UNUSUALLY LARGE AMOUNT of food (AND doing some of the things we talked about to keep from gaining weight around the same time). During ANY of these times, did eating LARGE AMOUNTS of food (AND doing some of the things we talked about to keep from gaining weight) . . .  Make you very upset?  Interfere with your normal daily activities?  Cause any serious problems getting along with other people – like arguing with your friends, family, people at work or anyone else?  Cause any serious problems doing the things you were supposed to do – like working, doing your schoolwork, or taking care of your home or family? |
| *Binge-Eating Disorder* | | |
| A. Report recurrent binge eating |  | See above section |
| B. Episodes associated with three or more of the following:   1. Eating much more rapidly than usual 2. Eating until feeling uncomfortably full 3. Eating large amounts of food when no feeling physically hungry 4. Eating alone because of being embarrassed by how much one is eating 5. Feeling disgusted with oneself, depressed, or very guilty after overeating | N18Q3C  N18Q3D  N18Q3E  N18Q3F  N18Q3G | During ANY time like this when you ate an UNUSUALLY LARGE AMOUNT of food, did you . . .  Find that you ate much more quickly than usual?  Find that you ate until you felt uncomfortably full?  Eat an UNUSUALLY LARGE AMOUNT of food even though you weren’t hungry?  Eat alone because you might be embarrassed by how much you were eating?  Feel disgusted with yourself, depressed or very guilty about eating so much?   - Respond “YES” to three out of the five questions |
| C. Marked distress regarding binge eating is present | N18Q27A^6^ | During ANY of those times when you ate an UNUSUALLY LARGE AMOUNT of food did this make you upset?   - Respond “YES” |
| D. Binge eating is not associated with regular use of inappropriate compensatory behaviors (e.g., laxative use, purging, fasting, excessive exercise) and does not occur exclusively during the course of anorexia or bulimia nervosa | N18Q26 | Were there EVER ANY OTHER times lasting at least 3 months when you ate LARGE AMOUNTS of food at least once a week WITHOUT doing any of the things you mentioned to keep from gaining weight?   - Respond “YES” or missing response^7^ |
| Age of onset | N18Q28B | About how old were you the FIRST time you BEGAN to eat LARGE AMOUNTS of food on average at least once a week for at least 3 months? |
| Age at the most recent episode | N18Q31R | How old were you the MOST RECENT time you BEGAN to eat LARGE AMOUNTS of food? |
| Duration of the most recent episode | N18Q33AR | How long did (this/your) MOST RECENT time last when you ate LARGE AMOUNTS of food (in months)?  Our operationalization of “12-months” diagnosis^3^   - Difference between age of onset and current age is 1 year or less. - Difference between current age and age at the most recent episode plus duration of the most recent episode is 1 year or less. - If we cannot calculate one of these variables, it was treated as no12-months diagnosis |
| Associated impairments | N18Q27A  N18Q27B  N18Q27C  N18Q27D | Now I’d like to ask you about some other things that might have happened to you when you were eating an UNUSUALLY LARGE AMOUNT of food. During ANY of these times, did eating LARGE AMOUNTS of food . . .  Make you very upset?  Interfere with your normal daily activities?  Cause any serious problems getting along with other people – like arguing with your friends, family, people at work or anyone else?  Cause any serious problems doing the things you were supposed to do – like working, doing your schoolwork, or taking care of your home or family? |

*Notes*:

^1^ The answer to this question was not included in the NESARC-III database. This is likely because the “lower than 85% weight” criterion is no longer used for AN in the *DSM-5*.

^2^ We selected BMI < 18.5 as a cut-off as it is a widely used convention for “normal” BMI.

^3^ There was also an additional question, “Did you restrict the amount of food that you ate in order to lose weight BEFORE you weighed (weight in N17Q1)?” (N17Q4B). We did *not* use this variable to categorize AN due to potential ambiguities (for example, a person might have lost weight due to a medical reason and then started to restrict once they achieved low weight). Clinically and diagnostically, we interpret the AN criterion A to capture or focus on restriction of food intake during the time of low weight.

^4^ N17Q6C & N17Q6D required reverse coding to reflect AN pathology.

^5^ NESARC-III included a question about whether the onset of EDs was in the past 12 months or whether the most recent episode began to occur in the past 12 months. We note that this variable alone cannot not indicate whether an individual had ED in the past 12 months as the most recent episode could begin to occur prior to the past 12 months and he/she still was experiencing the symptoms at the time of interview. We therefore took the categorization approach described above.

^6^ For one individual, we used N18Q7A instead of N18Q27A. This case responded “YES” to N18Q7A but “NO” to N18Q27A, but did not meet criteria for BN.

^7^ Our close inspection of the data indicated that close to 90% of individuals who were categorized as having lifetime BED either by the NESARC-III or by our coding are missing this variable. Of those with this response coded, 25 answered “NO” or “UNKNOWN”. These individuals were also missing information such as age of onset, the most recent episode, and associated impairment (because a “NO” or “UNKNOWN” response led to skipping the rest of BED-related questions). Although these individuals reported positive responses to all other BED criteria, we chose to take a conservative approach, and therefore did *not* categorize these individuals as meeting BED criteria, resulting in *N* = 318 (see Udo & Grilo, 2018, for sensitivity analyses).

*** Link to the actual survey question: <https://www.niaaa.nih.gov/research/nesarc-iii/questionnaire> (Sections 17 & 18 are related with EDs).
