# Supplementary material for: Suicide attempts in US adults with lifetime DSM-5 eating disorders
Source: BMC Med. 2019 Jun 25;17:120. doi: 10.1186/s12916-019-1352-3 (PMC6591971; doi:10.1186/s12916-019-1352-3)
Supplement: Supplementary file 2 — Table S2. Self-reported history of suicidal attempts (SAs) by lifetime history of DSM-5 anorexia nervosa (AN), bulimia nervosa (BN), binge-eating disorder (BED) (with application of hierarchical rule AN>BN>BED), and no specific eating disorder (ED) diagnosis. (DOCX 20 kb) [file 12916_2019_1352_MOESM2_ESM.docx]

**Table S2**. Self-Reported History of Suicidal Attempts (SAs) by Lifetime History of *DSM-5* Anorexia Nervosa (AN), Bulimia Nervosa (BN), Binge-Eating Disorder (BED) (with application of hierarchical rule AN>BN>BED), and No Specific Eating Disorder (ED) diagnosis.

|  | AN  (*n* = 276) | BN  (*n* = 77) | BED  (*n* = 247) | No specific ED diagnosis  (*n* = 35,571) |
| --- | --- | --- | --- | --- |
| Reporting a history of suicidal attempts | | | | |
| % (SE)  *n*  *(population estimates)* | 24.9 (3.12) ^a^  60  (97,031) | 18.6 (5.06) ^a^  19  (25,623) | 17.0 (2.67) ^a^  39  (79,206) | 4.9 (017)  1877  (11,905,675) |
| AORs (95% CIs) | 5.58 ^‡^  (3.93-7.93) | 3.13 ^‡^  (1.54-6.36) | 3.54 ^‡^  (2.39-5.24) | (reference) |
| The number of SAs ^1^ |  |  |  |  |
| Total sample | 0.51 (0.10) ^‡^ | 0.31 (0.15) | 0.39 (0.10) ^‡^ | 0.09 (0.004) |
| With SA history | 2.18 (0.39) | 1.92 (0.49) | 2.45 (0.38) | 1.78 (0.06) |
| Age of first attempt ^2^ | 23.3 (1.60) | 24.6 (1.95) | 25.5 (1.80) | 23.8 (0.30) |

*Notes*. Calculations of adjusted odds ratios (AORs) and 95% confidence intervals (CIs), means and associated standard errors included adjustments for sex, age, income, education, and race/ethnicity. All analyses were adjusted for the NESARC complex survey design.

^1^ = statistical analyses were based on log-transformed variables due to distribution properties. ^2^ = analysis only included individuals with SA history. ^‡^ = significantly different at *p* < .01. ^a^ = significantly different from no history of specific ED at *p* < .05 based on or comparison of cells (38, 39) .
