# Supplementary material for: Suicide attempts in US adults with lifetime DSM-5 eating disorders
Source: BMC Med. 2019 Jun 25;17:120. doi: 10.1186/s12916-019-1352-3 (PMC6591971; doi:10.1186/s12916-019-1352-3)
Supplement: Supplementary file 3 — Table S3. Comparison of self-reported history of suicidal attempts (SAs) between respondents meeting more than one lifetime eating disorder (ED) diagnosis and no history of specific ED diagnosis. (DOCX 19 kb) [file 12916_2019_1352_MOESM3_ESM.docx]

**Table S3**. Comparison of Self-Reported History of Suicidal Attempts (SAs) between Respondents Meeting More Than One Lifetime Eating Disorder (ED) diagnosis and no history of specific ED diagnosis.

|  | Multiple ED diagnoses^1^  (*n* = 73) | No specific ED diagnosis  (*n* = 35,571) |
| --- | --- | --- |
| Reporting a history of suicidal attempts | | |
| % (SE)  *n*  *(population estimates)* | 41.5 (6.73)  27  (26,134) | 4.9 (0.17)  1877  (11,905,675) |
| AORs (95% CIs) | 10.63 ^‡^ (5.92-19.10) | (reference) |
| The number of SAs ^2^ |  |  |
| Total sample | 1.15 (0.34) ^‡^ | 0.09 (0.004) |
| With SA history | 2.99 (0.77) | 1.78 (0.06) |
| Age of first attempt ^3^ | 23.4 (2.34) | 23.8 (0.40) |

*Notes*. Calculations of adjusted odds ratios (AORs) and 95% confidence intervals (CIs), means and associated standard errors included adjustments for sex, age, income, education, and race/ethnicity. All analyses were adjusted for the NESARC complex survey design.

^1^ = multiple ED diagnoses were defined as meeting more than one lifetime ED diagnosis; ^2^ = statistical analyses were based on log-transformed variables due to distribution properties. ^3^ = analysis only included individuals with SA history. ^‡^ = significantly different at *p* < .01.
